# Supplementary material for: Resolving the full spectrum of human genome variation using Linked-Reads
Source: Genome Res. 2019 Apr;29(4):635–45. doi: 10.1101/gr.234443.118 (PMC6442396; doi:10.1101/gr.234443.118)
Supplement: Supplemental Material [file supp_gr.234443.118_Supplemental_longranger-2.2.zip › longranger-2.2.tar.bz2!/lr2.2-public/lib/stan/cmdstan/stan/doc/api/html/namespacestan.html]

Stan: stan Namespace Reference


|  |  |
| --- | --- |
|  | Stan  2.5.0  probability, sampling & optimization |


- Main Page
- Related Pages
- Namespaces
- Classes
- Files

- Namespace List
- Namespace Members

All Classes Namespaces Files Functions Variables Typedefs Enumerations Enumerator Friends Macros Pages

Namespaces |
Classes |
Functions |
Variables

stan Namespace Reference

Probability, optimization and sampling library.
More...

|  |  |
| --- | --- |
| Namespaces | |
|  | agrad |
|  | Function gradients via reverse-mode automatic differentiation. |
|  | |
|  | common |
|  | |
|  | gm |
|  | Directed graphical model compiler. |
|  | |
|  | io |
|  | Input/output utilities. |
|  | |
|  | json |
|  | |
|  | math |
|  | Matrices and templated mathematical functions. |
|  | |
|  | mcmc |
|  | Markov chain Monte Carlo samplers. |
|  | |
|  | memory |
|  | (Expert) Allocate, free, and manage underlying system memory. |
|  | |
|  | model |
|  | For compiling models. |
|  | |
|  | optimization |
|  | |
|  | prob |
|  | Templated probability distributions. |
|  | |

|  |  |
| --- | --- |
| Classes | |
| struct | error\_index |
|  | |
| struct | is\_constant |
|  | Metaprogramming struct to detect whether a given type is constant in the mathematical sense (not the C++ `const` sense). More... |
|  | |
| struct | is\_constant\_struct |
|  | Metaprogram to determine if a type has a base scalar type that can be assigned to type double. More... |
|  | |
| struct | is\_constant\_struct< std::vector< T > > |
|  | |
| struct | is\_constant\_struct< Eigen::Matrix< T, R, C > > |
|  | |
| struct | is\_constant\_struct< Eigen::Block< T > > |
|  | |
| struct | is\_vector |
|  | |
| struct | is\_vector< const T > |
|  | |
| struct | is\_vector< std::vector< T > > |
|  | |
| struct | is\_vector< Eigen::Matrix< T, Eigen::Dynamic, 1 > > |
|  | |
| struct | is\_vector< Eigen::Matrix< T, 1, Eigen::Dynamic > > |
|  | |
| struct | is\_vector< Eigen::Block< T > > |
|  | |
| struct | scalar\_type |
|  | Metaprogram structure to determine the base scalar type of a template argument. More... |
|  | |
| struct | size\_of\_helper |
|  | |
| struct | size\_of\_helper< T, true > |
|  | |
| struct | scalar\_type< Eigen::Matrix< T, Eigen::Dynamic, Eigen::Dynamic > > |
|  | |
| struct | scalar\_type< T \* > |
|  | |
| struct | is\_vector\_like |
|  | |
| struct | is\_vector\_like< T \* > |
|  | |
| struct | is\_vector\_like< const T > |
|  | |
| struct | is\_vector\_like< Eigen::Matrix< T, Eigen::Dynamic, Eigen::Dynamic > > |
|  | |
| class | VectorView |
|  | VectorView is a template metaprogram that takes its argument and allows it to be used like a vector. More... |
|  | |
| class | VectorView< const T, is\_array, throw\_if\_accessed > |
|  | VectorView that has const correctness. More... |
|  | |
| class | VectorView< const double, false, false > |
|  | |
| class | DoubleVectorView |
|  | DoubleVectorView allocates double values to be used as intermediate values. More... |
|  | |
| class | DoubleVectorView< true, false > |
|  | |
| class | DoubleVectorView< true, true > |
|  | |
| struct | return\_type |
|  | Metaprogram to calculate the base scalar return type resulting from promoting all the scalar types of the template parameters. More... |
|  | |
| struct | is\_fvar |
|  | |
| struct | is\_fvar< stan::agrad::fvar< T > > |
|  | |
| struct | is\_var |
|  | |
| struct | is\_var< stan::agrad::var > |
|  | |
| struct | contains\_fvar |
|  | Metaprogram to calculate the base scalar return type resulting from promoting all the scalar types of the template parameters. More... |
|  | |
| struct | is\_var\_or\_arithmetic |
|  | |
| struct | scalar\_type\_pre |
|  | Metaprogram structure to determine the type of first container of the base scalar type of a template argument. More... |
|  | |
| class | VectorViewMvt |
|  | |
| class | VectorViewMvt< const T, is\_array, throw\_if\_accessed > |
|  | VectorViewMvt that has const correctness. More... |
|  | |

|  |  |
| --- | --- |
| Functions | |
| template<typename T > | |
| T | get (const T &x, size\_t n) |
|  | |
| template<typename T > | |
| T | get (const std::vector< T > &x, size\_t n) |
|  | |
| template<typename T , int R, int C> | |
| T | get (const Eigen::Matrix< T, R, C > &m, size\_t n) |
|  | |
| template<typename T > | |
| size\_t | length (const T &) |
|  | |
| template<typename T > | |
| size\_t | length (const std::vector< T > &x) |
|  | |
| template<typename T , int R, int C> | |
| size\_t | length (const Eigen::Matrix< T, R, C > &m) |
|  | |
| template<typename T > | |
| size\_t | size\_of (const T &x) |
|  | |
| template<typename T1 , typename T2 > | |
| size\_t | max\_size (const T1 &x1, const T2 &x2) |
|  | |
| template<typename T1 , typename T2 , typename T3 > | |
| size\_t | max\_size (const T1 &x1, const T2 &x2, const T3 &x3) |
|  | |
| template<typename T1 , typename T2 , typename T3 , typename T4 > | |
| size\_t | max\_size (const T1 &x1, const T2 &x2, const T3 &x3, const T4 &x4) |
|  | |
| template<typename T > | |
| size\_t | length\_mvt (const T &) |
|  | |
| template<typename T , int R, int C> | |
| size\_t | length\_mvt (const Eigen::Matrix< T, R, C > &) |
|  | |
| template<typename T , int R, int C> | |
| size\_t | length\_mvt (const std::vector< Eigen::Matrix< T, R, C > > &x) |
|  | |
| template<typename T1 , typename T2 > | |
| size\_t | max\_size\_mvt (const T1 &x1, const T2 &x2) |
|  | |
| template<typename T1 , typename T2 , typename T3 > | |
| size\_t | max\_size\_mvt (const T1 &x1, const T2 &x2, const T3 &x3) |
|  | |
| template<typename T1 , typename T2 , typename T3 , typename T4 > | |
| size\_t | max\_size\_mvt (const T1 &x1, const T2 &x2, const T3 &x3, const T4 &x4) |
|  | |

|  |  |
| --- | --- |
| Variables | |
| const std::string | MAJOR\_VERSION = "2" |
|  | Major version number for Stan package. More... |
|  | |
| const std::string | MINOR\_VERSION = "5" |
|  | Minor version number for Stan package. More... |
|  | |
| const std::string | PATCH\_VERSION = "0" |
|  | Patch version for Stan package. More... |
|  | |

## Detailed Description

Probability, optimization and sampling library.

## Function Documentation

template<typename T >

|  |  |  |  |
| --- | --- | --- | --- |
| T stan::get | ( | const T & | *x*, |
|  |  | size\_t | *n* |
|  | ) |  |  |

inline

Definition at line 143 of file traits.hpp.

template<typename T >

|  |  |  |  |
| --- | --- | --- | --- |
| T stan::get | ( | const std::vector< T > & | *x*, |
|  |  | size\_t | *n* |
|  | ) |  |  |

inline

Definition at line 147 of file traits.hpp.

template<typename T , int R, int C>

|  |  |  |  |
| --- | --- | --- | --- |
| T stan::get | ( | const Eigen::Matrix< T, R, C > & | *m*, |
|  |  | size\_t | *n* |
|  | ) |  |  |

inline

Definition at line 151 of file traits.hpp.

template<typename T >

|  |  |  |  |  |  |
| --- | --- | --- | --- | --- | --- |
| size\_t stan::length | ( | const T & |  | ) |  |

Definition at line 159 of file traits.hpp.

template<typename T >

|  |  |  |  |  |  |
| --- | --- | --- | --- | --- | --- |
| size\_t stan::length | ( | const std::vector< T > & | *x* | ) |  |

Definition at line 163 of file traits.hpp.

template<typename T , int R, int C>

|  |  |  |  |  |  |
| --- | --- | --- | --- | --- | --- |
| size\_t stan::length | ( | const Eigen::Matrix< T, R, C > & | *m* | ) |  |

Definition at line 167 of file traits.hpp.

template<typename T >

|  |  |  |  |  |  |
| --- | --- | --- | --- | --- | --- |
| size\_t stan::length\_mvt | ( | const T & |  | ) |  |

Definition at line 561 of file traits.hpp.

template<typename T , int R, int C>

|  |  |  |  |  |  |
| --- | --- | --- | --- | --- | --- |
| size\_t stan::length\_mvt | ( | const Eigen::Matrix< T, R, C > & |  | ) |  |

Definition at line 566 of file traits.hpp.

template<typename T , int R, int C>

|  |  |  |  |  |  |
| --- | --- | --- | --- | --- | --- |
| size\_t stan::length\_mvt | ( | const std::vector< Eigen::Matrix< T, R, C > > & | *x* | ) |  |

Definition at line 570 of file traits.hpp.

template<typename T1 , typename T2 >

|  |  |  |  |
| --- | --- | --- | --- |
| size\_t stan::max\_size | ( | const T1 & | *x1*, |
|  |  | const T2 & | *x2* |
|  | ) |  |  |

Definition at line 191 of file traits.hpp.

template<typename T1 , typename T2 , typename T3 >

|  |  |  |  |
| --- | --- | --- | --- |
| size\_t stan::max\_size | ( | const T1 & | *x1*, |
|  |  | const T2 & | *x2*, |
|  |  | const T3 & | *x3* |
|  | ) |  |  |

Definition at line 198 of file traits.hpp.

template<typename T1 , typename T2 , typename T3 , typename T4 >

|  |  |  |  |
| --- | --- | --- | --- |
| size\_t stan::max\_size | ( | const T1 & | *x1*, |
|  |  | const T2 & | *x2*, |
|  |  | const T3 & | *x3*, |
|  |  | const T4 & | *x4* |
|  | ) |  |  |

Definition at line 206 of file traits.hpp.

template<typename T1 , typename T2 >

|  |  |  |  |
| --- | --- | --- | --- |
| size\_t stan::max\_size\_mvt | ( | const T1 & | *x1*, |
|  |  | const T2 & | *x2* |
|  | ) |  |  |

Definition at line 575 of file traits.hpp.

template<typename T1 , typename T2 , typename T3 >

|  |  |  |  |
| --- | --- | --- | --- |
| size\_t stan::max\_size\_mvt | ( | const T1 & | *x1*, |
|  |  | const T2 & | *x2*, |
|  |  | const T3 & | *x3* |
|  | ) |  |  |

Definition at line 582 of file traits.hpp.

template<typename T1 , typename T2 , typename T3 , typename T4 >

|  |  |  |  |
| --- | --- | --- | --- |
| size\_t stan::max\_size\_mvt | ( | const T1 & | *x1*, |
|  |  | const T2 & | *x2*, |
|  |  | const T3 & | *x3*, |
|  |  | const T4 & | *x4* |
|  | ) |  |  |

Definition at line 590 of file traits.hpp.

template<typename T >

|  |  |  |  |  |  |
| --- | --- | --- | --- | --- | --- |
| size\_t stan::size\_of | ( | const T & | *x* | ) |  |

Definition at line 186 of file traits.hpp.

## Variable Documentation

const std::string stan::MAJOR\_VERSION = "2"

Major version number for Stan package.

Definition at line 9 of file version.hpp.

const std::string stan::MINOR\_VERSION = "5"

Minor version number for Stan package.

Definition at line 12 of file version.hpp.

const std::string stan::PATCH\_VERSION = "0"

Patch version for Stan package.

Definition at line 15 of file version.hpp.


---

[ Stan Home Page ]

*© 2011–2014,
Stan Development Team.*
